# Validity and reliability study of the Turkish version of the self-efficacy for managing chronic disease 6-item scale

**DOI:** 10.3906/sag-1910-13

**Published:** 2020-08-26

**Authors:** Kübra İNCİRKUŞ, Nursen ÖZKAN NAHCİVAN

**Affiliations:** 1 Department of Public Health Nursing, Faculty of Health Sciences, Trakya University, Edirne Turkey; 2 Department of Public Health Nursing, Florence Nightingale Faculty of Nursing, İstanbul University–Cerrahpaşa, İstanbul Turkey

**Keywords:** Chronic disease, self-efficacy, reliability, validity

## Abstract

**Background/aim:**

The measurement of self-efficacy is an important step toward improving chronic disease management, enhancing adherence to treatment, and assessing patients with chronic diseases. The purpose of this study was to assess the validity and reliability of the Turkish version of the Self-Efficacy for Managing Chronic Disease 6-Item Scale.

**Materials and methods:**

In this methodological study, the sample consisted of 211 patients receiving chronic disease care in 2 family health centers in İstanbul, Turkey. Data were analyzed with descriptive statistics, exploratory and confirmatory factor analyses, concurrent validity, Cronbach’s alpha, item-total correlation, and test-retest reliability.

**Results:**

Most of the sample were women (72%), primary school graduates (41.7%), hypertensive (68.7%), and had comorbidities (68.2%). The mean age of the sample was 60.5 ± 10.9. The scale mean was 5.66 ± 2.28. Cronbach’s alpha reliability was 0.90. Item-total correlations were between 0.64–0.85 and test-retest reliability was high (R = 0.95, P < 0.001). A positive, significant correlation was found in concurrent validity. According to the results of factor analysis, the scale had a 2-dimensional structure.

**Conclusion:**

The Turkish version of the Self-Efficacy for Managing Chronic Disease 6-Item Scale is a valid and reliable instrument to assess perceived the self-efficacy level of patient with chronic disease.

## 1. Introduction

Chronic diseases constitute a large part of diseases that cause mortality in Turkey as well as all over the world, and the burden of chronic diseases will widely increase [1 **–** 3]. People with chronic diseases should switch to healthy lifestyle behavior and maintain it, and take up new tasks every day [4]. Management of chronic diseases includes more than treatment of the disease; it also includes strengthening the patient through various teaching methods to gain self-management skills [5,6]. Self-management is a part of daily life for people who have lived for many years with chronic diseases, and each day they make self-management decisions, or take problem-solving actions throughout their lives [4,7]. Self-management skills also consist of finding and utilizing resources, collaboration between patient and health care providers, and taking action with self-efficacy [4]. 

Self-efficacy is defined as a person’s beliefs about their capabilities for carrying out an action plan, tackling challenges, and making judgments in making a specific action successful. It affects behavior choice and the environment [8,9]. Self-efficacy is a central component, a significant outcome variable, and an important indicator for deciding health education programs, a major part of behavior change processes, and a precondition for a successful self-management of chronic diseases [7,10 **–** 12]. High self-efficacy is related to better health status, the improvement of health behavior, motivation, problem-solving and complex thinking skills, healing, decision-making, psychological well-being, and fewer emergency department visits [4,8,13 **–** 15]. 

The measurement of self-efficacy is an important step toward improving chronic disease management, enhancing adherence to the recommended treatment, and assessing patients in terms of chronic diseases [11,16,17]. Determining the level of a patient’s perceived self-efficacy provides assistance in deciding on suitable interventions for increased self-care, planning patient education programs, predicting the level of a patient’s intent, readiness, the support needed for behavioral change and maintenance, and evaluating the impact of interventions [11,16,17]. There are various tools for measuring self-efficacy in the literature. Many of these tools assess general or condition/disease-specific self-efficacy [18], such as the Arthritis Self-Efficacy Scale [19], Diabetes Management Self-Efficacy Scale [20,21], Medication Adherence Self-Efficacy Scale [22], Generalized Perceived Self-Efficacy Scale [23,24], Self-Efficacy Scale [25], General Self-Eﬃcacy Scale [26], COPD Self-Efficacy Scale [27], and Medication Adherence Self-Efficacy Scale-Short Form [28]. On the other hand, these available instruments evaluate generic or just a single disease or condition-specific self-efficacy level. The Self-Efficacy for Managing Chronic Disease 6-Item Scale is a brief and effective tool that directly measures the self-efficacy for chronic diseases. In this context, the purpose of this study was to assess the validity and reliability of the Turkish version of the Self-Efficacy for Managing Chronic Disease 6-Item Scale. In this way, cross-cultural studies or comparisons regarding self-efficacy in chronic disease management may be possible.

## 2. Materials and methods

### 2.1. Study design, setting and sample 

This methodological study was conducted as a preliminary study of an experimental study aimed to evaluate the results of the motivational interview-based self-management support program for hypertensive patients in 2 family health centers in İstanbul, Turkey. Accordingly, the study was carried out in densely populated and centrally located family health centers where individuals of different socioeconomic levels receive health services. Family health centers are 1 of the units in which preventive and therapeutic health care services (such as maternal-child health, immunization, family planning services, and diagnosing, treating, monitoring, and following up for chronic diseases) are provided for the community. These centers are places where people with chronic diseases are diagnosed, treated and provided with home visits and other primary health care services. Additionally, in such places, rehabilitation and coordination with secondary and tertiary health services are carried out, and individual health records are kept and monitored [29]. 

The sample of this study includes 211 patients with chronic diseases. For methodological research, the sample size is recommended to be at least 5 **–** 10 times more than the number of the items of the scale [30,31]. Purposeful sampling method was used in the study and the sampling criteria were determined as follows: being 18 years of age or older, having 1 or more chronic disease for 6 months or longer, having no hearing-visual impairments or mental problems, and agreeing to participate in the study. Individuals who were diagnosed with Alzheimer’s, a psychiatric disease, had malignancy, or refused to participate were excluded from the study. The Turkish version of the Standardized Mini-Mental State Examination was used to assess the cognitive status of individuals who were older than 65 years. Patients who scored above the threshold (23/24) set for the Turkish community were included in the study [32]. Since the list of individuals with chronic disease was not available at the time of the study, the sample consisted of patients in the waiting rooms of the centers. Within the scope of the research, a similar number of individuals were included in the study from each family health centers. Fifty-four percent (n = 114) of the sample was selected from the first, and 46% (n = 97) from the second family health center.

### 2.2. Instruments

The Interview Form, the Self-Efficacy for Managing Chronic Disease 6-Item Scale, and the General Perceived Self-Efficacy Scale were the data collection tools of this study. The interview form consisted of questions including the sociodemographic and health characteristics of the patients such as age, gender, education level, marital status, chronic disease, and comorbidity status. Information regarding the current chronic diseases and comorbidities were acquired by asking the patients directly. The questions were formed as follows: “What is your current diagnosed chronic disease?” and, “Do you have any other diagnosed chronic disease other than the one you mentioned?”

#### 2.2.1. The Self-Efficacy for Managing Chronic Disease 6-Item Scale 

The Self-Efficacy for Managing Chronic Disease 6-Item Scale was developed by Lorig and colleagues in English in 2001 [15]. It is easy to use and an effective tool for evaluating the self-efficacy level of patients with chronic diseases. The scale is rated on a 10-point scale ranging from “not at all confident” to “totally confident”. The score for the scale is the mean of the 6 items, and high scores indicate high self-efficacy. If more than 1 response is given to an item and the items are consecutive, the lower score is included in the calculation. If the 2 given responses are not consecutive, this item is excluded from the calculation. In order for the scale to be calculated, there must be at least 4 items answered Self-Management Resource Center. Self-Efficacy for Managing Chronic Disease 6-Item Scale. [online]. Website u2994 [accessed November 11, 2019].. There is a high internal consistency (α = 0.91) and the mean of original scale is 5.17 ± 2.22 [15].

#### 2.2.2. The General Perceived Self-Efficacy Scale 

The General Perceived Self-Efficacy Scale was used for examining the concurrent validity of the Self-Efficacy for Managing Chronic Disease 6-Item Scale. The General Perceived Self-Efficacy Scale, consisting of 10 items, is a valid and reliable measurement tool in Turkish [23,24]. The scale measures the level of generalized perceived self-efficacy. Cronbach’s alpha of the scale was 0.89, and Pearson’s product-moment correlation was between 0.64–0.78, factor loading of the scale’s items changed from 0.64 to 0.79 in one factor structure, and test-retest correlation was 0.83 [24]. In this study, Cronbach’s alpha was 0.94, item-total score correlation was between 0.51–0.82, test-retest correlation was 0.96 (P < 0.001) for the General Perceived Self-Efficacy Scale. 

### 2.3. Data collection

Data were collected via face-to-face interviews in the counseling room in family health centers. The patients were at first called by phone and the ones who accepted to participate in the study were invited to the family health center and interviews were initiated. Each interview lasted 15 min on average. Patients’ questions were also answered during the interview. Four weeks after the first assessments, the sampled patients were called again, and all assessments were repeated for test-retest reliability. Because of self-efficacy is a changeable psychological condition, a shorter (<2 weeks) retest period is recommended [11]. In the recent validity and reliability studies of various self-efficacy scales, this period was between 2–4 weeks [24 **–** 27,33]. In this study, the retest period was set as 4 weeks. 

### 2.4. Translation and cultural adaptation 

The World Health Organization guideline suggests that 4 steps (forward translation, expert panel and back-translation, pretesting and cognitive interviewing, final version) are needed to achieve translation and adaptation of different language versions of the English instrument World Health Organization. Process of translation and adaptation of instruments. [online]. Website u2998 [accessed November 11, 2019].. The English-to-Turkish translation of the scale was independently done by the researchers. Two English teachers working in the university and a professional translator translated the scale from English to Turkish. Then the scale was brought into a single form by the researchers. Back-translation from Turkish to English was independently done by 2 native English speakers living in Turkey for many years. After the back-translation, the scale items were revised in terms of grammar, clarity, cultural properties, and it was made available for an expert panel. The final Turkish translation was presented to a total of 12 healthcare experts working in various fields related to chronic diseases (a diabetes specialist nurse, an internal diseases specialist, and 10 nursing academicians with expertise in chronic disease management) for a language and content validity check. For content validity of the scale, the experts evaluated each scale item with a 4-point Likert-type scale (1 not relevant, 4 highly relevant). Items given 1 and 2 points were rearranged by the researcher. The Content Validity Index (CVI) score of the scale was calculated by the proportion of items that were scored 3 or 4 points by the experts and if the score was 0.80 or higher, it was considered acceptable [34,35]. In the direction of expert evaluation, to shorten the questions and increase clarity of the scale, the commonly used expression of “How confident are you...” was subtracted from all of the questions and added to the top of the scale. After the pilot test of the scale was performed on 10 people with chronic diseases, it was ready to be utilized for psychometric assessments [Appendix 1].

### 2.5. Ethical considerations

After receiving institutional permission, the study was approved by the Ethics Committee of Zeynep Kamil Hospital (Ref. no. 045, date 05/04/2013). All patients were informed, and written consents were obtained before data collection. 

### 2.6. Data analysis 

Statistical analyses were carried out using SPSS 21.0 (IBM Corp., Armonk, NY, USA) and LISREL 8.80 (Lincolnwood, IL, USA) software. Descriptive data were expressed via mean ± standard deviation (X ± SD), minimums, maximums, and percentages (%). Validity data were evaluated with concurrent validity, exploratory (including Varimax rotation with Kaiser Normalization), and confirmatory factor analysis. Data suitability for factor analysis was analyzed using the Kaiser-Meyer-Olkin (KMO) value and Bartlett’s test [30,36]. The items with factor loadings 0.40 and above were included in the factor structure [36]. The reliability of the scale was evaluated with Cronbach’s alpha, item-total correlation, and test-retest reliability. In evaluation of the results, the item-total score correlation was expected to be 0.30 or more, and the retest correlation and alpha coefficient 0.70 or more [36 **–** 38]. The comparisons between scale mean and sample characteristics data were evaluated via regression analysis. Concurrent validity and test-retest reliability were evaluated via Pearson’s correlation analysis. The significance level was considered as P < 0.05. 

## 3. Results

### 3.1. Sample characteristics 

Most of the participants with a mean age of 60.5 ± 10.9 (min 33, max 85) were women (72%), primary school graduates (41.7%), hypertensive (68.7%), diabetic (15.6%), asthmatic (7.1%), and had comorbidities (68.2%) (Table 1). The average years of having one or more chronic disease were 10.8 ± 9.8 (min 1, max 53). Mean score of the 6-Item Scale and some characteristics of the participants (age, sex, education, chronic diseases, and comorbidity) were compared with regression analysis. It was showed that the self-efficacy scores were lower in people with lower educational status (β = 0.435, t = 6.892, P < 0.001) and hypertension (β = **–** 0.155, t = **–** 2.478, P = 0.014). There was no statistically significant difference between mean score and age, sex, comorbidity, and other chronic diseases (P ˃ 0.05). 

**Table 1:** General characteristics of the participants (N = 211).

Characteristics		n	(%)
Gender	Female	152	(72.0)
	Male	59	(28.0)
Education level	Literate	23	(10.9)
	Primary School	88	(41.7)
	Secondary School	19	(9.0)
	High School	44	(20.9)
	University	37	(17.5)
Marital status	Married	149	(70.6)
	Single	62	(29.4)
Chronic diseases	Hypertension	145	(68.7)
	Diabetes	33	(15.6)
	Asthma	15	(7.1)
	Other (COPD, depression etc.)	18	(8.6)
Comorbidity	Yes	144	(68.2)
	No	67	(31.8)

### 3.2. Validity

In this study, the validity of the scale was evaluated via Content Validity Index (CVI), concurrent validity, factor analysis and the CVI score of the scale was 0.81. For concurrent validity, a positive, significant, and medium correlation was found between the Self-Efficacy for Managing Chronic Disease 6-Item Scale and the General Perceived Self-Efficacy Scale (Pearson’s correlation, r = 0.54, P < 0.001). 

Before factor analysis, Kaiser-Meyer-Olkin (KMO) value and Bartlett’s test results were assessed. KMO value was 0.794 and Bartlett’s test was found significant (χ2 = 1136.546, P < 0.001). The results of exploratory factor analysis were showed that the scale had 2-factor structure, and 98.02% of total variance was explained by 2 factors. Factor 1 explained 94.50% and factor 2 explained 3.52% of total variance. Factor loadings of the scale were between 0.83–0.95 for exploratory factor analysis and between 0.77–0.98 for confirmatory factor analysis. Eigenvalues, % of variance, factor loadings for exploratory (EFA) and confirmatory (CFA) factor analysis, and Cronbach’s Alpha values for each factor are shown in Table 2. Chi-square value was significant (χ2 = 21.52, df = 8, P = 0.006, RMSEA = 0.090). The other fit index values of the scale were; IFI (incremental fit index) = 0.99, GFI (goodness of fit index) = 0.97, NNFI (nonnormed fit index) = 0.98, CFI (comparative fit index) = 0.99, SRMR (standardized root-mean square residual) = 0.027. Standardized confirmatory factor analysis path diagram is presented in Figure.

**Table 2:** Table 2. Eigenvalues, % of variance, factor loadings for exploratory (EFA), and confirmatory (CFA) factor analysis and Cronbach’s alpha values for each factor.

Scale Items	Eigenvalues	% of Variance	Factor loadings for EFA		Factor loadings for CFA		Cronbach’salpha values
			Factor 1	Factor 2	Factor 1	Factor 2	
1	3.992	66.529	0.83	0.21	0.77	-	0.92
2	1.184	19.732	0.88	0.19	0.85	-	
3	0.357	5.949	0.89	0.21	0.88	-	
4	0.256	4.268	0.86	0.35	0.94	-	
5	0.145	2.423	0.24	0.95	-	0.95	0.96
6	0.066	1.100	0.26	0.95	-	0.98	

**Figure:**
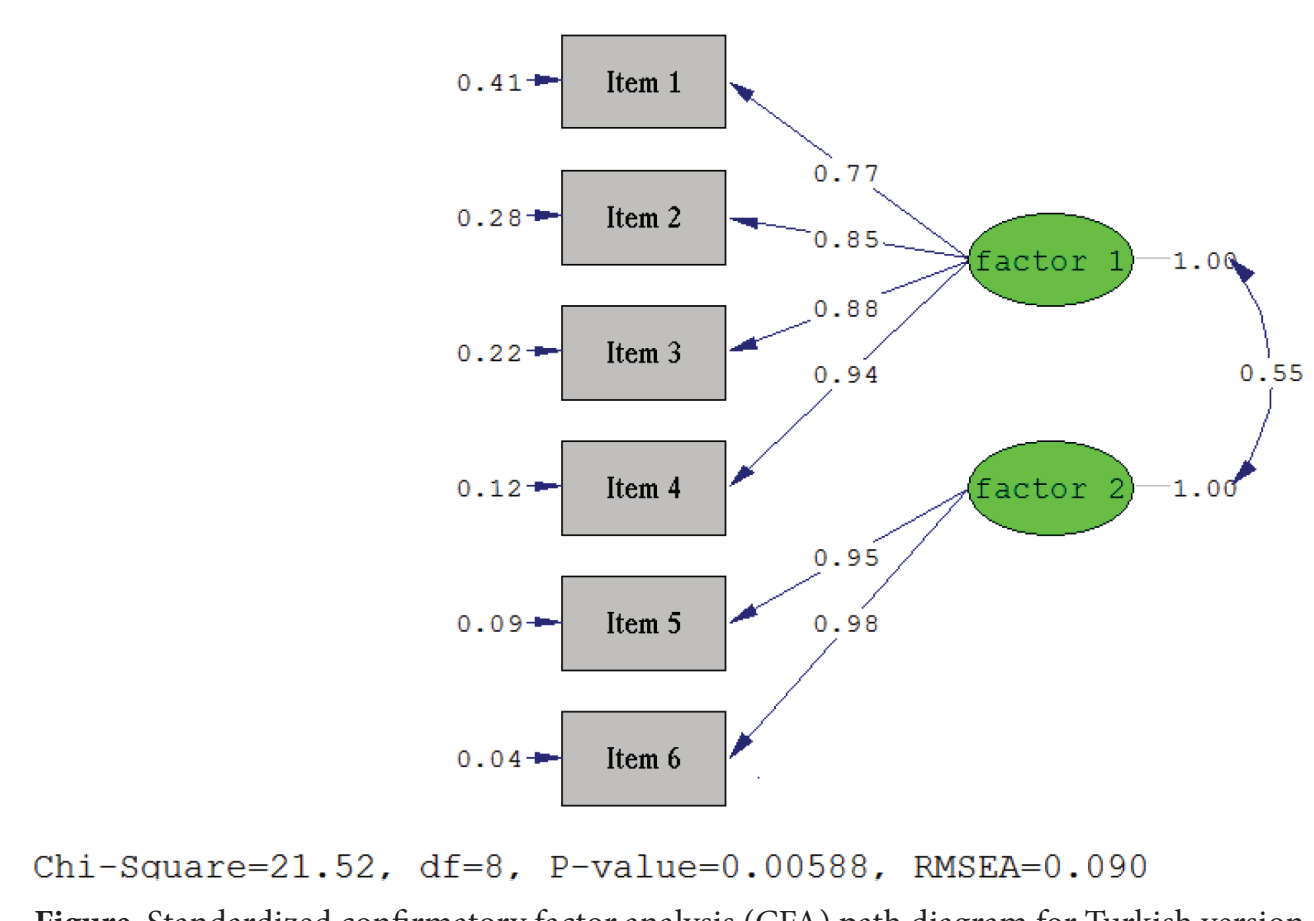
Standardized confirmatory factor analysis (CFA) path diagram for Turkish version of the Self-Efficacy for Managing Chronic Disease 6-Item Scale.

### 3.3. Reliability

The item-total correlations, the Cronbach’s alpha coefficient, and test-retest correlations were evaluated in this study. Item-total correlations were ranged from 0.64 to 0.85 (P < 0.001) and the scale mean was 5.66 ± 2.28 (Table 3). Cronbach’s alpha reliability of the scale was 0.90. In order to examine the stability in terms of time, the test-retest reliability was implemented with 116 patients who filled in the scale before. During the course of 1 month (4 weeks) the test-retest reliability was high (Pearson’s correlation, r = 0.95, P < 0.001).

**Table 3:** Mean, standard deviation (SD), minimum-maximum (Min-Max), and item-total correlations of the scale items (N = 211).

Scale Items	Mean (± SD)	Min-max	Item-totalcorrelations
1. How confident are you that you can keep the fatigue caused by your disease from interfering with the things you want to do?	5.39 (± 2.27)	2–10	0.70
2. How confident are you that you can keep the physical discomfort or pain of your disease from interfering with the things you want to do?	5.57 (± 2.74)	2–10	0.75
3. How confident are you that you can keep the emotional distress caused by your disease from interfering with the things you want to do?	5.20 (± 2.99)	2–10	0.76
4. How confident are you that you can keep any other symptoms or health problems you have from interfering with the things you want to do?	5.39 (± 2.74)	2–10	0.85
5. How confident are you that you can do the different tasks and activities needed to manage your health condition so as to reduce you need to see a doctor?	6.25 (± 2.76)	2–10	0.64
6. How confident are you that you can do things other than just taking medication to reduce how much your illness affects your everyday life?	6.14 (± 2.79)	2–10	0.65
Total scale item mean (± SD), min-max	5.66 (± 2.28), 2–10		

## 4. Discussion

Being an important outcome variable, self-efficacy is a part of the long-term behavioral change process and is a prerequisite for successful chronic disease management [10 **–** 12]. Self-efficacy assessment is an increasingly critical concept in planning and evaluating the self-management programs in chronic diseases, determining individual differences among patients, and estimating important health outcomes [11]. Especially on scales that assess the abstract concepts such as self-efficacy, validity, and reliability value is becoming more important in the adaptations of the scales developed in different languages and cultures. Validity shows how accurately a tool measures something [34,36]. Validity data were evaluated with content validity index, exploratory and confirmatory factor analysis, and concurrent validity. The language equivalence of the translated version of the scale from English to Turkish was evaluated with the Content Validity Index. The CVI score is expected to be above 0.80 [31,34 **–** 36], so it was adequate (0.81) in this study. 

The scale showed a 2-dimensional structure in both factor analyses. Although the result of factor analysis has not been given in the original study [15], the scale has been found to have 1-dimensional structure in other studies except the Chinese study [16,39,40]. In our study, similar to the Chinese study [16], items 5 and 6 have been gathered under the factor 2. Hu and colleagues [16] reported 2 possible reasons for this situation. First, while the first 4 items were stressing the psychological attitude, the last 2 items were stressing behavioral attitude. Hu and colleagues stated as the second reason that the results could be specific to the sample of their research. In the sample of their study, it is pointed out that the lack of age and literacy alternatives, the high number of women, and participants who might tend to give the desired results in face-to-face interviews may have affected the study results [16]. In this study, the majority of the sample was female and the use of face-to-face interviews to collect data may have led to similar results. 

Concurrent validity evaluates an instrument’s validity by comparing it to a valid scale or test [36]. In the German study [39], the same scale was used for concurrent validity and a good correlation was found (r = 0.578, P < 0.001). Concurrent validity was evaluated with Hospital Anxiety and Depression Scale in the Chinese study [16] and with Health Education Impact Questionnaire in the French study [33]. It was reported that the 6-item scale showed a significant correlation with Hospital Anxiety and Depression Scale (r = **–** 0.30, P < 0.001) and Health Education Impact Questionnaire (r = 0.49, P < 0.001) [16,33]. For concurrent validity, a positive and significant correlation was found in this study (r = 0.54, P < 0.001). The results obtained in this study were similar to the results of studies carried out in other languages. A significant and good correlation was found between the Self-Efficacy for Managing Chronic Disease 6-Item Scale and the General Perceived Self-Efficacy Scale *.*

The reliability of the scale was evaluated with the Cronbach’s alpha coefficient, item-total statistics, test-retest correlations in this study. Publications related to research methodologies indicate that test-retest reliability and Cronbach’s alpha values should be higher than 0.70 [36 **–** 38]. Cronbach’s alpha reliability was between 0.88–0.93 in the validity and reliability studies of the scale in the other languages [15,16,33,39,40]. Alpha coefficient of the study was found to be high (α = 0.90) in our study, and this value was similar to the results of the other validity and reliability studies. Although the test-retest period (4 weeks) took longer than other validity and reliability studies of self-efficacy scales [24 **–** 27,33], test-retest reliability was found to be very high (r = 0.95, P < 0.001) in this study (n = 116). The test-retest reliability was 0.78 [16] and 0.82 [33] in other validity and reliability studies of the 6-item scale. Item-total correlations were between 0.64 **–** 0.85 (P < 0.001) and this value was considered good compared to the suggested value (>0.30) [36,38]. It was also similar to the results of other studies [16,33,39,40]. 

Although this study focused on examining the psychometric properties of the Self-Efficacy for Managing Chronic Disease 6-Item Scale, the mean self-efficacy score was compared to the sociodemographic and health characteristics of the total sample. It was showed that self-efficacy scores were lower in people who were hypertensive and with lower educational status; however, no correlation was found between age, sex, number of comorbidities, other chronic diseases, and scale scores in this study. Similar to these results, in the study of Dongbo et al. [41] it was reported that higher education is associated with higher self-efficacy and better health outcomes. In another study in which sociodemographic data and scale scores were compared, a negative correlation was found between age, sex, number of comorbidities, and the self-efficacy scores, but no correlation was found between educational status and the self-efficacy scores [39]. 

Although significant and positive results were obtained, the patients constituting the sample of the study were selected from only 2-family health centers and most of these patients had hypertension and diabetes. Although this situation reduced the strength of the sample that represents patients with different chronic health problems, these diseases were stated within the first 3 diseases in the original study as well as other validity and reliability studies. 

In conclusion, the Turkish version of the Self-Efficacy for Managing Chronic Disease 6-Item Scale is a reliable and validated instrument to assess the self-efficacy level perceived by patients with chronic disease. Self-efficacy is a prerequisite and predictor of successful chronic disease self-management [10,39]. By using the 6-item Scale in the assessment of individuals who need to improve their self-efficacy, the development of effective practices that increase the level of self-efficacy and improvement of chronic disease self-management will be provided [16,40]. It is also stated that the scale can be used to assess patient participation as well as the difference according to initial assessment of self-efficacy [40]. The results of our study showed good external validity, high internal consistency and test-retest reliability, and 2-dimensional structure. Further studies are recommended in a larger sample group representing individuals with chronic diseases who use the scale of health professionals in the management of chronic diseases.

## Acknowledgements

We sincerely thank the patients who participated in this study and the health workers for their support.

## Conflict of Interest

The authors declare that there is no conflict of interest. All listed authors meet the authorship criteria and they are in agreement with the content of the manuscript.

Supplementary Materials
