# Supplementary material for: Validity and reliability study of the Turkish version of the self-efficacy for managing chronic disease 6-item scale
Source: Turk J Med Sci. 2020 Aug 26;50(5):1254–61. doi: 10.3906/sag-1910-13 (PMC7491285; doi:10.3906/sag-1910-13)
Supplement: Supplementary file 1 — Supplementary Materials [file turkjmedsci-50-1254-sup001.pdf]

Appendix 1. Turkish version of the Self-Efficacy for Managing Chronic Disease 6-Item Scale [Kronik Hastalık Yönetimi İçin 6 Maddelik Öz-Etkililik Ölçeği].

Kronik hastalığı olan bireyler için öz-yönetim günlük hayatın bir parçasıdır. Aşağıda kronik hastalığınızın yönetiminde karşılaşabileceğiniz bazı faaliyetler verilmiştir. Size daha iyi bir sağlık hizmeti sunabilmemiz için, bu faaliyetleri yapma konusunda ne kadar emin olduğunuzu öğrenmek istiyoruz. Lütfen aşağıda yer alan her bir ifadeyi dikkatlice okuyarak yanıt vermeye çalışınız. Şu anda düzenli olarak yapabileceğiniz durumlar için **kendinize olan güveninize karşılık gelen** uygun sayıyı seçiniz ve seçtiğiniz sayıyı yuvarlak içine alınız. Lütfen her bir ifadenin cevaplanmış olmasına dikkat ediniz.

Hastalığınızın neden olduğu sorunları yönetmede kendinize ne kadar güveniyorsunuz?

1. Yorgunluğumun yapmak istediklerimi etkilemesini önleyebilirim.	**Hiç emin değilim** 1 2 3 4 5 6 7 8 9 10 **Tamamen eminim**
2. Fiziksel rahatsızlık ve ağrımın yapmak istediklerimi etkilemesini önleyebilirim.	**Hiç emin değilim** 1 2 3 4 5 6 7 8 9 10 **Tamamen eminim**
3. Duygusal sıkıntımın yapmak istediklerimi etkilemesini önleyebilirim.	**Hiç emin değilim** 1 2 3 4 5 6 7 8 9 10 **Tamamen eminim**
4. Sağlık problemleri ve diğer belirtilerimin yapmak istediklerimi etkilemesini önleyebilirim.	**Hiç emin değilim** 1 2 3 4 5 6 7 8 9 10 **Tamamen eminim**
5. Doktora daha az gitmek için, sağlığım ile ilgili çeşitli görev ve aktiviteleri yapabilirim.	**Hiç emin değilim** 1 2 3 4 5 6 7 8 9 10 **Tamamen eminim**
6. Hastalığımın günlük yaşamıma olan etkilerini azaltmak için, ilaç almaktan başka şeyler de yapabilirim.	**Hiç emin değilim** 1 2 3 4 5 6 7 8 9 10 **Tamamen eminim**
